# 1-(4-Methyl­phen­yl)-1*H*-1,2,3,4-tetra­zole

**DOI:** 10.1107/S1600536812000797

**Published:** 2012-01-14

**Authors:** Kwan Baek, D. Gayathri, Vivek K. Gupta, Rajni Kant, Yeon Tae Jeong

**Affiliations:** a Department of Image Science and Engineering, Pukyong National University, Busan 608 739, Republic of Korea; b Department of Physics, Dr. M.G.R Educational and Research Institute, Dr. M.G.R University, Maduravoyal, Chennai 600 095, India; c X-ray Crystallography Laboratory, Post Graduate Department of Physics & Electronics, University of Jammu, Jammu Tawi 180 006, India

## Abstract

In the title compound, C_8_H_8_N_4_, the dihedral angle between the tetra­zole and benzene rings is 21.6 (1)°. An inter­molecular C—H⋯π inter­action is observed.

## Related literature

For background to and applications of tetra­zole derivatives, see: Singh *et al.* (1980 ▶); Brown (1967 ▶); Ostrovskii *et al.* (1999 ▶). For the synthesis, see: Aridoss & Laali (2011 ▶). For related structures, see: Matsunaga *et al.* (1999 ▶); Lyakhov *et al.* (2000 ▶).
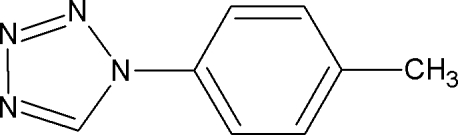


## Experimental

### 

#### Crystal data

C_8_H_8_N_4_
*M* *_r_* = 160.18
Monoclinic, 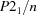

*a* = 9.8352 (13) Å
*b* = 5.7244 (6) Å
*c* = 14.4190 (19) Å
β = 96.285 (12)°
*V* = 806.92 (17) Å^3^
*Z* = 4
Mo *K*α radiation
μ = 0.09 mm^−1^
*T* = 293 K
0.3 × 0.2 × 0.1 mm

#### Data collection

Oxford Diffraction Xcalibur Sapphire3 diffractometer
Absorption correction: multi-scan (*CrysAlis PRO*; Oxford Diffraction, 2010 ▶) *T*_min_ = 0.762, *T*_max_ = 1.000
14618 measured reflections
1419 independent reflections
936 reflections with *I* > 2σ(*I*)
*R*_int_ = 0.054

#### Refinement

*R*[*F* ^2^ > 2σ(*F* ^2^)] = 0.067
*wR*(*F* ^2^) = 0.211
*S* = 1.05
1419 reflections
110 parameters
H-atom parameters constrained
Δρ_max_ = 0.21 e Å^−3^
Δρ_min_ = −0.25 e Å^−3^

### 

Data collection: *CrysAlis PRO* (Oxford Diffraction, 2010 ▶); cell refinement: *CrysAlis PRO*; data reduction: *CrysAlis RED* (Oxford Diffraction, 2010 ▶); program(s) used to solve structure: *SHELXS97* (Sheldrick, 2008 ▶); program(s) used to refine structure: *SHELXL97* (Sheldrick, 2008 ▶); molecular graphics: *PLATON* (Spek, 2009 ▶); software used to prepare material for publication: *PLATON*.

## Supplementary Material

Crystal structure: contains datablock(s) I, global. DOI: 10.1107/S1600536812000797/is5047sup1.cif

Structure factors: contains datablock(s) I. DOI: 10.1107/S1600536812000797/is5047Isup2.hkl

Supplementary material file. DOI: 10.1107/S1600536812000797/is5047Isup3.cml

Additional supplementary materials:  crystallographic information; 3D view; checkCIF report

## Figures and Tables

**Table 1:** Hydrogen-bond geometry (Å, °) *Cg* is the centroid of the C2–C7 ring.

*D*—H⋯*A*	*D*—H	H⋯*A*	*D*⋯*A*	*D*—H⋯*A*
C6—H6⋯*Cg*^i^	0.93	2.89	3.630 (3)	138

Symmetry code: (i) 
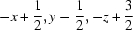
.

**Fig. 1.**
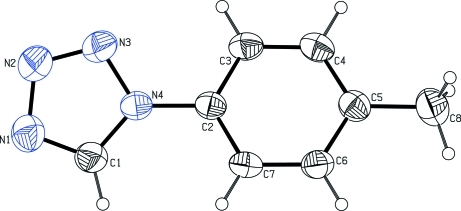
The molecular structure of the title compound, showing 30% probability displacement ellipsoids.

## supplementary crystallographic information

### Comment

Tetrazoles are an important functional group with wide range of applications (Aridoss & Laali, 2011). They function as ligands in coordination chemistry, in medicinal chemistry as a metabolically stable surrogate for a carboxylic acid group (Singh *et al.*, 1980) and in materials science applications including propellants (Brown, 1967) and explosives (Ostrovskii *et al.*, 1999).

Bond lengths and bond angles are comparable with the similar crystal structures (Matsunaga *et al.*, 1999; Lyakhov *et al.*, 2000). The tetrazole and benzene rings are planar with maximum deviations of 0.006 (2) and 0.011 (2) Å (r.m.s. deviations of the rings being 0.005 and 0.008 Å), respectively. The two rings are not coplanar with the dihedral angle being 21.6 (1)°. Methyl carbon atom lies 0.014 (5) Å from the plane of the phenyl ring. Bond distances C1—N1 [1.304 (4) Å] and N2—N3 [1.289 (4) Å] indicate the consistence of the formation of double bonds.

### Experimental

The title compound was synthesized from the known procedure reported elsewhere (Aridoss & Laali, 2011). Fine white diffraction quality crystals were obtained from the slow evaporation of its solution in ethanol.

### Refinement

All H atoms were refined using a riding model, with C—H = 0.93 Å (aromatic) or 0.96 Å (methyl), and with *U*_iso_(H) = 1.2*U*_eq_(C) or 1.5*U*_eq_(methyl C).

### Crystal data

**Table:**

C_8_H_8_N_4_	*F*(000) = 336
*M**_r_* = 160.18	*D*_x_ = 1.319 Mg m^−^^3^
Monoclinic, *P*2_1_/*n*	Mo *K*α radiation, λ = 0.71073 Å
Hall symbol: -P 2yn	Cell parameters from 3527 reflections
*a* = 9.8352 (13) Å	θ = 3.6–29.2°
*b* = 5.7244 (6) Å	µ = 0.09 mm^−^^1^
*c* = 14.4190 (19) Å	*T* = 293 K
β = 96.285 (12)°	Plate, white
*V* = 806.92 (17) Å^3^	0.3 × 0.2 × 0.1 mm
*Z* = 4	

### Data collection

**Table:**

Oxford Diffraction Xcalibur Sapphire3 diffractometer	1419 independent reflections
Radiation source: fine-focus sealed tube	936 reflections with *I* > 2σ(*I*)
graphite	*R*_int_ = 0.054
Detector resolution: 16.1049 pixels mm^-1^	θ_max_ = 25.0°, θ_min_ = 3.8°
ω scans	*h* = −11→11
Absorption correction: multi-scan (*CrysAlis PRO*; Oxford Diffraction, 2010)	*k* = −6→6
*T*_min_ = 0.762, *T*_max_ = 1.000	*l* = −17→17
14618 measured reflections	

### Refinement

**Table:**

Refinement on *F*^2^	Primary atom site location: structure-invariant direct methods
Least-squares matrix: full	Secondary atom site location: difference Fourier map
*R*[*F*^2^ > 2σ(*F*^2^)] = 0.067	Hydrogen site location: inferred from neighbouring sites
*wR*(*F*^2^) = 0.211	H-atom parameters constrained
*S* = 1.05	*w* = 1/[σ^2^(*F*_o_^2^) + (0.1133*P*)^2^ + 0.1542*P*] where *P* = (*F*_o_^2^ + 2*F*_c_^2^)/3
1419 reflections	(Δ/σ)_max_ < 0.001
110 parameters	Δρ_max_ = 0.21 e Å^−^^3^
0 restraints	Δρ_min_ = −0.25 e Å^−^^3^

### Special details

**Table:**

Geometry. All e.s.d.'s (except the e.s.d. in the dihedral angle between two l.s. planes) are estimated using the full covariance matrix. The cell e.s.d.'s are taken into account individually in the estimation of e.s.d.'s in distances, angles and torsion angles; correlations between e.s.d.'s in cell parameters are only used when they are defined by crystal symmetry. An approximate (isotropic) treatment of cell e.s.d.'s is used for estimating e.s.d.'s involving l.s. planes.
Refinement. Refinement of *F*^2^ against ALL reflections. The weighted *R*-factor *wR* and goodness of fit *S* are based on *F*^2^, conventional *R*-factors *R* are based on *F*, with *F* set to zero for negative *F*^2^. The threshold expression of *F*^2^ > σ(*F*^2^) is used only for calculating *R*-factors(gt) *etc*. and is not relevant to the choice of reflections for refinement. *R*-factors based on *F*^2^ are statistically about twice as large as those based on *F*, and *R*- factors based on ALL data will be even larger.

### Fractional atomic coordinates and isotropic or equivalent isotropic displacement parameters (Å^2^)

**Table:**

	*x*	*y*	*z*	*U*_iso_*/*U*_eq_	
C1	0.7153 (3)	0.0846 (5)	0.8500 (2)	0.0726 (9)	
H1	0.7271	−0.0729	0.8372	0.087*	
C2	0.4656 (3)	0.0810 (4)	0.87347 (17)	0.0540 (7)	
C3	0.3730 (3)	0.1934 (5)	0.92335 (18)	0.0635 (8)	
H3	0.3959	0.3336	0.9537	0.076*	
C4	0.2465 (3)	0.0950 (5)	0.9274 (2)	0.0672 (9)	
H4	0.1838	0.1718	0.9605	0.081*	
C5	0.2090 (3)	−0.1143 (5)	0.88414 (19)	0.0622 (8)	
C6	0.3039 (3)	−0.2219 (5)	0.8337 (2)	0.0634 (8)	
H6	0.2811	−0.3625	0.8036	0.076*	
C7	0.4315 (3)	−0.1253 (4)	0.82696 (18)	0.0607 (8)	
H7	0.4932	−0.1980	0.7918	0.073*	
C8	0.0709 (3)	−0.2228 (6)	0.8897 (2)	0.0850 (10)	
H8A	0.0534	−0.2328	0.9538	0.127*	
H8B	0.0692	−0.3767	0.8631	0.127*	
H8C	0.0017	−0.1283	0.8558	0.127*	
N1	0.8114 (3)	0.2424 (5)	0.85255 (19)	0.0841 (9)	
N2	0.7502 (3)	0.4443 (5)	0.8748 (2)	0.0874 (9)	
N3	0.6234 (3)	0.4089 (4)	0.8848 (2)	0.0826 (9)	
N4	0.5985 (2)	0.1809 (4)	0.86824 (14)	0.0580 (7)	

### Atomic displacement parameters (Å^2^)

**Table:**

	*U*^11^	*U*^22^	*U*^33^	*U*^12^	*U*^13^	*U*^23^
C1	0.077 (2)	0.0625 (18)	0.081 (2)	0.0000 (16)	0.0255 (17)	−0.0042 (15)
C2	0.0584 (16)	0.0504 (15)	0.0528 (15)	0.0089 (11)	0.0041 (12)	0.0054 (11)
C3	0.079 (2)	0.0557 (16)	0.0568 (17)	0.0051 (14)	0.0129 (14)	−0.0082 (13)
C4	0.071 (2)	0.0697 (19)	0.0636 (18)	0.0125 (15)	0.0181 (15)	−0.0055 (14)
C5	0.0616 (18)	0.0644 (17)	0.0609 (17)	0.0079 (13)	0.0075 (14)	0.0067 (13)
C6	0.0678 (18)	0.0531 (16)	0.0686 (18)	0.0045 (13)	0.0046 (14)	−0.0049 (13)
C7	0.0661 (19)	0.0524 (15)	0.0635 (18)	0.0138 (13)	0.0073 (14)	−0.0052 (12)
C8	0.066 (2)	0.101 (3)	0.088 (2)	−0.0003 (17)	0.0104 (17)	−0.0046 (19)
N1	0.0828 (19)	0.086 (2)	0.087 (2)	−0.0101 (15)	0.0223 (15)	0.0017 (14)
N2	0.085 (2)	0.0721 (18)	0.106 (2)	−0.0069 (15)	0.0121 (16)	0.0022 (15)
N3	0.082 (2)	0.0569 (16)	0.108 (2)	0.0002 (13)	0.0060 (16)	−0.0030 (13)
N4	0.0649 (15)	0.0519 (13)	0.0568 (14)	0.0047 (11)	0.0048 (11)	0.0029 (10)

### Geometric parameters (Å, °)

**Table:**

C1—N1	1.304 (4)	C5—C8	1.504 (4)
C1—N4	1.326 (3)	C6—C7	1.385 (4)
C1—H1	0.9300	C6—H6	0.9300
C2—C3	1.380 (4)	C7—H7	0.9300
C2—C7	1.381 (4)	C8—H8A	0.9600
C2—N4	1.436 (3)	C8—H8B	0.9600
C3—C4	1.373 (4)	C8—H8C	0.9600
C3—H3	0.9300	N1—N2	1.357 (4)
C4—C5	1.382 (4)	N2—N3	1.288 (4)
C4—H4	0.9300	N3—N4	1.345 (3)
C5—C6	1.389 (4)		
			
N1—C1—N4	110.3 (3)	C5—C6—H6	119.1
N1—C1—H1	124.8	C2—C7—C6	118.8 (3)
N4—C1—H1	124.8	C2—C7—H7	120.6
C3—C2—C7	120.8 (3)	C6—C7—H7	120.6
C3—C2—N4	119.9 (2)	C5—C8—H8A	109.5
C7—C2—N4	119.2 (2)	C5—C8—H8B	109.5
C4—C3—C2	119.0 (3)	H8A—C8—H8B	109.5
C4—C3—H3	120.5	C5—C8—H8C	109.5
C2—C3—H3	120.5	H8A—C8—H8C	109.5
C3—C4—C5	122.3 (2)	H8B—C8—H8C	109.5
C3—C4—H4	118.8	C1—N1—N2	105.0 (3)
C5—C4—H4	118.8	N3—N2—N1	110.6 (2)
C4—C5—C6	117.4 (3)	N2—N3—N4	107.0 (2)
C4—C5—C8	122.1 (3)	C1—N4—N3	107.1 (2)
C6—C5—C8	120.5 (3)	C1—N4—C2	131.2 (2)
C7—C6—C5	121.7 (3)	N3—N4—C2	121.7 (2)
C7—C6—H6	119.1		
			
C7—C2—C3—C4	1.0 (4)	C1—N1—N2—N3	−0.1 (4)
N4—C2—C3—C4	180.0 (2)	N1—N2—N3—N4	−0.6 (4)
C2—C3—C4—C5	0.7 (4)	N1—C1—N4—N3	−1.2 (3)
C3—C4—C5—C6	−1.4 (4)	N1—C1—N4—C2	180.0 (3)
C3—C4—C5—C8	179.3 (3)	N2—N3—N4—C1	1.1 (3)
C4—C5—C6—C7	0.3 (4)	N2—N3—N4—C2	−180.0 (2)
C8—C5—C6—C7	179.7 (3)	C3—C2—N4—C1	157.9 (3)
C3—C2—C7—C6	−2.0 (4)	C7—C2—N4—C1	−23.2 (4)
N4—C2—C7—C6	179.0 (2)	C3—C2—N4—N3	−20.7 (4)
C5—C6—C7—C2	1.3 (4)	C7—C2—N4—N3	158.2 (2)
N4—C1—N1—N2	0.9 (3)		

### Hydrogen-bond geometry (Å, °)

**Table:**

*Cg* is the centroid of the C2–C7 ring.

**Table:**

*D*—H···*A*	*D*—H	H···*A*	*D*···*A*	*D*—H···*A*
C6—H6···Cg^i^	0.93	2.89	3.630 (3)	138

Symmetry codes: (i) −*x*+1/2, *y*−1/2, −*z*+3/2.
